# Photophysical and Electrochemical Studies of Multinuclear Complexes of Iron(II) with Acetate and Extended Conjugated N-Donor Ligands

**DOI:** 10.1155/2015/860537

**Published:** 2015-03-23

**Authors:** Norbani Abdullah, Suhana Mohd Said, Anita Marlina, Muhamad Faris Roslan, Afiq Azil, Abdul Rahman Nordin

**Affiliations:** ^1^Department of Chemistry, Faculty of Science, University of Malaya, 50603 Kuala Lumpur, Malaysia; ^2^Department of Electrical Engineering, Faculty of Engineering, University of Malaya, 50603 Kuala Lumpur, Malaysia

## Abstract

A dimeric iron(II) complex, *trans*-[Fe_2_(CH_3_COO)_4_(L1)_2_] (**1**), and a trinuclear iron(II) complex, [Fe_3_(CH_3_COO)_4_(H_2_O)_4_(L2)] (**2**), were studied as potential dye-sensitised solar cell materials. The structures of both complexes were deduced by a combination of instrumental analyses and molecular modelling. Variable-temperature magnetic susceptibility data suggested that **1** was made up of 56.8% high-spin (HS) and 43.2% low-spin (LS) Fe(II) atoms at 294 K and has a moderate antiferromagnetic interaction (*J* = −81.2 cm^−1^) between the two Fe(II) centres, while **2** was made up of 27.7% HS and 72.3% LS Fe(II) atoms at 300 K. The optical band gaps (*E*_*o*_) for **1** were 1.9 eV (from absorption spectrum) and 2.2 eV (from fluorescence spectrum), electrochemical bandgap (*E*_e_) was 0.83 eV, excited state lifetime (*τ*) was 0.67 ns, and formal redox potential (*E*′(Fe^III^/Fe^II^)) was +0.63 V. The corresponding values for **2** were 3.5 eV (from absorption spectrum), 1.8 eV (from fluorescence spectrum), 0.69 eV, 2.8 ns, and +0.41 V.

## 1. Introduction

Polypyridyl complexes of Fe(II) are currently attracting the attention of researchers as potential photosensitizers in dye-sensitized solar cells (DSSC) [1–3]. These low-spin complexes have similar structures as the corresponding Ru(II) complexes [4–9] but are much cheaper, easier to prepare, and less toxic.

We noted that the Fe(II) complexes reported as potential DSSC materials were mostly mononuclear. Hence, we focused our research on multinuclear Fe(II) complexes, especially involving *π*-conjugated ligands, as they are expected to have better photosensitization (especially absorption of lower photonic energy) and redox properties.

**Figure 1:**
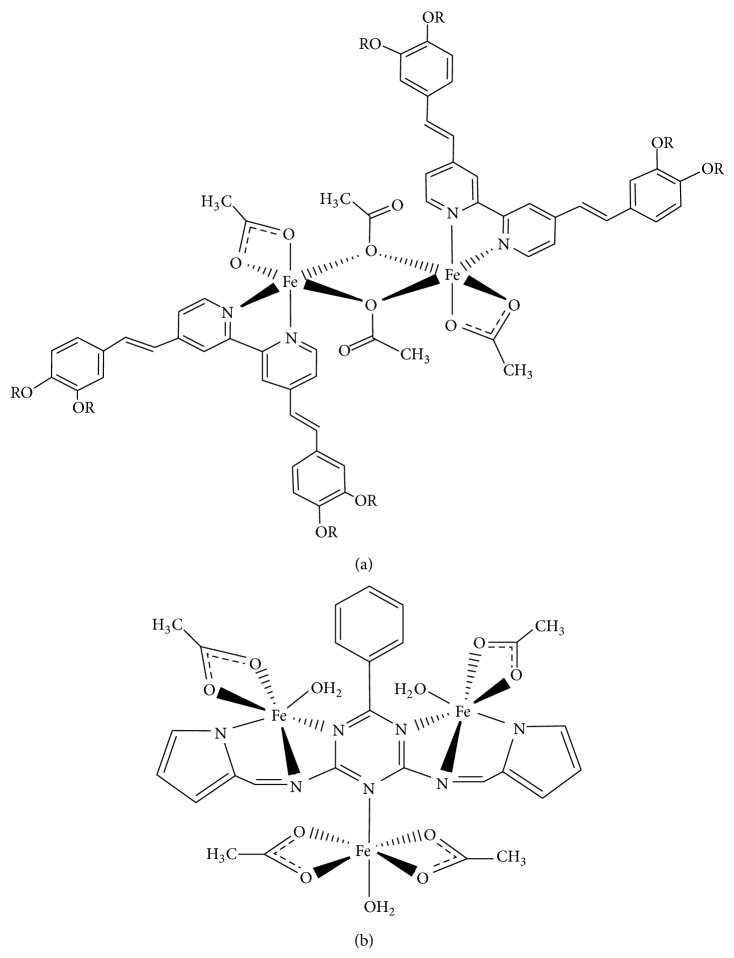
Proposed structures of (a)** 1** (R = CH_3_(CH_2_)_13_ and (b)** 2**.

This paper reports the syntheses, structural deduction, and determinations of band gaps and excited state lifetimes of a dimeric iron(II) complex,* trans*-[Fe_2_(CH_3_COO)_4_(L1)_2_] (**1**) and a trinuclear iron(II) complex, [Fe_3_(CH_3_COO)_4_(H_2_O)_4_(L2)] (**2**) (Figure 1). L1 (4,4′-bis[3,4-bis(tetradecyloxy)styryl]-2,2′-bipyridine) was a bidentate N_2_-bipyridyl donor, while L2 was a multidentate N-donor dianion of a Schiff base, 6-phenyl-*N,N*′-bis-(1*H*-pyrrol-2-ylmethylene)-[1,3,5]-triazine-2,4-diamine. The magnetic data of both complexes are also presented to establish the spin states of the Fe(II) centres. The main objective of this paper was to establish a correlation between band gaps and excited state lifetimes with the nuclearity and spin states of a complex, and types of conjugated ligands (2,2′-bipyridine versus Schiff base).

## 2. Experimental Results

3,4-Bis(tetradecyloxy)benzaldehyde was a gift from Professor D. W. Bruce from the Department of Chemistry, University of York, United Kingdom. Other chemicals were commercially available and used as received. The elemental analyses (C, H, N) were carried out on a Thermo Finnigan Flash EA 1112. The ^1^H-NMR spectrum was recorded on a JEOL FT-NMR lambda 400 MHz spectrometer. The FTIR spectra were recorded for neat samples from 4000 cm^−1^ to 450 cm^−1^ on a Perkin-Elmer Frontier FTIR spectrometer equipped with a diamond attenuated total reflectance attachment. The UV-Vis spectra were recorded from 1000 nm to 400 nm on a Shimadzu UV-Vis-NIR 3600 spectrophotometer. The low-temperature magnetic susceptibilities were measured on a Quantum Design MPMS XL EverCool SQUID magnetometer at 1 Tesla and temperature range 300–2 K. The raw data was analysed using Microsoft Excel and IGOR Pro. The excitation and emission photoluminescence spectra were recorded on a PTI QuantaMaster 40 spectrofluorometer. The slit widths were set at a resolution of 10 nm for excitation and 5 nm for emission. The fluorescence life time measurement was performed on a PTI TimeMaster (TM-200) LED-based Strobe Lifetime spectrofluorometer using the stroboscopic technique. The observed fluorescence decay was analyzed using PTI Felix GX data acquisition and analysis software. Data was recorded in 100 ps time intervals from 50 ns to 70 ns observation window. The instrument response function was measured from the scattered light and estimated to be about 1.5 ns (full width at half maximum). The measured transients were fitted to multiexponential functions convoluted with the system response function. The fitting procedure was based on the Marquardt algorithm, where the experimental data were compared to a model decay convoluted with the IRF. The fit was judged by the value of the reduced *χ* ^2^. The cyclic voltammetric scans (CV) were recorded on a Gamry Instrument Reference 600 potentiostat/galvanostat/ZRA. The electrolyte was tetrabutylammonium tetrafluoroborate (TBATFB) (0.05 M), working electrode was glassy carbon, reference electrode was saturated calomel, and counter electrode was platinum wire. The initial and final voltages were 0 V, the potential range was +1.5 V to −1.5 V, and the scan rate was 100 mV s^−1^. The molarities of the samples were 0.005 M, and N_2_ was bubbled through the solutions prior to analyses.

### 2.1. Syntheses

#### 2.1.1. [3,4-Bis(tetradecyloxy)benzylidene]aniline

Aniline (1.08 g, 11.7 mmol) was added to a magnetically stirred hot ethanolic solution (75 cm^3^) of 3,4-bis(tetradecyloxy)benzaldehyde (5.02 g, 9.45 mmol), followed by a few crystals of* p*-toluenesulfonic acid. The reaction mixture was cooled to room temperature and left stirring overnight. The solvent was removed under reduced pressure, and the powder obtained was filtered and washed with ethanol. The yield was 5.6 g (98%).

#### 2.1.2. L1

[3,4-Bis(tetradecyloxy)benzylidene]aniline (5.00 g, 8.25 mmol) was dissolved in DMF (50 cm^3^) by heating at 50°C. 4,4′-Dimethyl-2,2′-bipyridine (0.76 g, 4.12 mmol) was added to the hot solution, and the reaction mixture was flushed with nitrogen. Potassium* t*-butoxide (4.04 g, 36.0 mmol) was added portionwise, and the reaction mixture was flushed again with nitrogen, heated to 80°C, and left stirring at this temperature for about 3 h. Hydrochloric acid (10%) was added to the cooled mixture until pH 7, followed by distilled water (200 cm^3^) and dichloromethane (300 cm^3^). The organic layer was washed with saturated sodium bicarbonate (200 cm^3^) and then with distilled water (200 cm^3^). It was dried over anhydrous sodium sulphate, and the solvent was removed under reduced pressure. The yield was 4.8 g (97%). ^1^H-NMR (400 MHz, CDCl_3_, *δ*/ppm): 0.88 (*t*, 12H), 1.26–1.60 (*m*, 88H), 1.85 (*m*, 8H), 4.1 (*m*, 8H), 6.91 (*d*, 2H; *J* = 12 Hz), 7.27 (*d*, 2H; *J* = 12 Hz), 7.31 (*d*, 2H), 7.58 (*s*, 2H), 8.22 (*s*, 2H), 8.34 (*s*, 2H), 7.53 (*d*, 2H), and 8.53 (*d*, 2H). Selected IR bands ($\overset{-}{\upsilon }$/cm^−1^) were 2917 vs, 2850 vs, 1589 s, 1576 s, 1512 s, 1467 m, 1435 s, 1386 m, 1270 vs, 1237 s, 1209 m, 1165 m, 1135 vs, 822 m, and 808 m.

#### 2.1.3. H_2_L2

An ethanolic solution of pyrrole-2-carboxaldehyde (11.69 g, 123.0 mmol) was added to an ethanolic suspension of 2,4-diamino-6-phenyl-1,3,5-triazine (11.52 g, 61.5 mmol). The mixture was refluxed in the presence of a few drops of glacial acetic acid for 2 h. The brownish solid formed was filtered from the hot reaction mixture, washed with cold ethanol, and dried in an oven at 80°C. The yield was 15.8 g (68%). ^1^H-NMR (DMSO-*d*_6_, *δ*/ppm) was 6.75 (*b*, 4H), 7.43–7.52 (*m*, 7H), and 8.23–8.25 (*d*, 4H). Selected IR bands ($\overset{-}{\upsilon }$/cm^−1^) were 3292 (w), 3137 (w), 1620 (s), 1536 (s), 1389 (s), and 1257 (w) cm^−1^.

#### 2.1.4. *Trans*-[Fe_2_(CH_3_COO)_4_(L1)_2_] (**1**)

[Fe(CH_3_COO)_2_] (0.26 g, 1.5 mmol) and ascorbic acid (0.43 g, 2.44 mmol) were dissolved in methanol (50 cm^3^) at room temperature, forming a black solution. A solution of L1 (1.75 g, 1.5 mmol) in chloroform (10 mL) was gradually added to the black solution. The reaction mixture was stirred at room temperature for 4 h, and then the solvents were evaporated off at room temperature. The dark purple powder formed was successively washed with distilled water, aqueous methanol (1 : 1), and methanol and dried in a warm oven for 1 h. The yield was 1.4 g (70%).* Anal.* Calc. for FeC_86_H_138_N_2_O_8_: C, 74.29; H, 10.48; N, 2.01%. Found: C, 74.96; H, 11.25; N, 1.84%. Selected IR bands ($\overset{-}{\upsilon }$/cm^−1^): 2917 (vs), 2849 (vs), 1686 (s), 1672 (m), 1596 (m), 1586 (m), 1511 (s), 1466 (m), 1390 (m), 1272 (vs), 1236 (m), 1165 (m), 1134 (vs), 1069 (m), 1053 (m), 1036 (m), 1018 (m), 1005 (m), 807 (s).

#### 2.1.5. [Fe_3_(CH_3_COO)_4_(H_2_O)_4_(L2)] (**2**)

[Fe(CH_3_COO)_2_] (0.96 g, 5.5 mmol) was added to an ethanolic suspension of H_2_L2 (1.89 g, 5.5 mmol), followed by about 0.1 g ascorbic acid as an antioxidant. The mixture was refluxed for 3 h, and the black solid formed was filtered off from the hot reaction mixture, washed with cold ethanol, and dried in an oven at 100°C. The yield was 2.8 g (97%).* Anal.* Calc. for Fe_3_C_27_H_29_N_7_O_12_: C, 39.8; H, 4.1, N, 12.0%. Found: C, 39.6; H, 3.8, N, 12.1%. Selected IR bands ($\overset{-}{\upsilon }$/cm^−1^) were 3296 (b), 1592 (w), 1532 (s), 1401 (s).

### 2.2. Computational Method

The first principle code DMol3 from Accelrys Material Studio (version 6.1), which employs the density functional theory (DFT) [10, 11], was used in optimization calculation and vibrational analysis. The electron correlation was treated by the spin polarized generalized gradient approximation (GGA) with Perdew, Burke, and Ernzerhof functional (PBE) [12]. The wave functions were represented by using a double-numerical-plus-polarization atomic orbital basis set. The geometry optimization was continued until the changes in energy and atomic displacement were less than 1.0 × 10^−5^ Hartree and 0.005 Å, respectively. The maximum force allowed was set at 0.002 Hartree Å^−1^. Dmol3 generated a Hessian matrix and this was then used to perform a frequency calculation. Harmonic vibrational frequencies were computed by diagonalizing the mass-weighted second-derivative matrix, *F* [13]. The elements of *F* were obtained by the following equation:

(1) $$\begin{array}{c} F_{ij}=\frac{1}{\sqrt{m_{i}m_{j}}}\frac{\partial^{2}E}{\partial q_{i}\cdot \partial q_{j}}, \end{array}$$

where *q*_*i*_ and *q*_*j*_ refer to the two Cartesian coordinates of atoms *i* and *j* and *m*_*i*_ and *m*_*j*_ are the masses of the respective atoms. The square roots of the eigenvalues of *F* are the harmonic frequencies and were used to verify the vibrational frequencies of the complexes. These results were then compared to the experimental results.

Dmol3 calculates the vibrational intensities from the atomic polar tensors (*A*), usually called Bohr effective charges. *A* is a second derivative energy with respect to the Cartesian coordinates and dipole moments

(2) $$\begin{array}{c} A_{i,j}=\frac{\partial E}{\partial q_{i}\partial \mu_{i}}. \end{array}$$

The intensity of a particular mode was calculated by squaring all transition moments of the mode and expressed in terms of the *A* matrix and eigenvectors of the mass-weighted Hessian. *F* is

(3) $$\begin{array}{c} I_{i}={\left(\underset{j,k}{\sum }F_{i,j}^{\prime }A_{j,k}\right)}^{2}, \end{array}$$

where *F*′ refers to the eigenvectors of the normal mode, *i*.

## 3. Results and Discussion

### 3.1. Syntheses and Structural Deduction

**Scheme 1:**
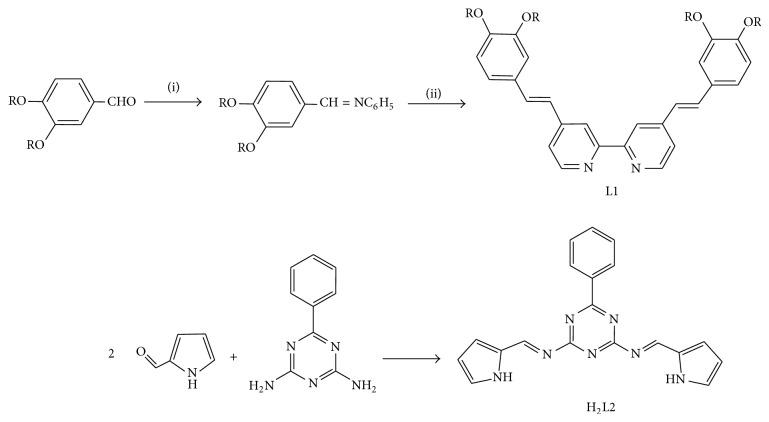
Steps for the syntheses of L1 (R = CH_3_(CH_2_)_13_) and H_2_L2. (i) Aniline,* p*-toluenesulfonic acid, EtOH, RT, 24 h; (ii) 4,4′-dimethyl-2,2′-bipyridine, KO^*t*^Bu, DMF, 80°C, 2 h.

The synthetic steps for the preparation of L1 and H_2_L2 are shown in Scheme 1.

The structural formulas of L1 and H_2_L2 were ascertained from ^1^H-NMR and FTIR spectroscopies (Section 2). From ^1^H-NMR spectroscopy, the *J* value for the vinylic H atoms in L1 was 12 Hz, indicating an *E* configuration for the ligand (as shown).

The dinuclear complex,* trans*-[Fe_2_(CH_3_COO)_4_(L1)_2_] (**1**), was obtained as dark purple powder in good yield (60%) from the reaction of L1 with [Fe(CH_3_COO)_2_] (mol ratio = 1 : 1). The powder was readily soluble in CH_2_Cl_2_, CHCl_3,_ and toluene. Its structural formula was proposed based on the CHN elemental analytic data and IR spectrum, which showed peaks for C=O at 1686 cm^−1^, ${\overset{-}{\upsilon }}_{\text{asym}}$COO at 1672 cm^−1^ and 1596 cm^−1^, ${\overset{-}{\upsilon }}_{\text{sym}}$COO at 1466 cm^−1^, and peaks for L1. The Δ values for CH_3_COO ligand ($\Delta ={\overset{-}{\upsilon }}_{\text{asym}}\text{COO}-{\overset{-}{\upsilon }}_{\text{sym}}\text{COO}$) were 130 cm^−1^ and 206 cm^−1^, which suggest bidentate chelating and monodentate bridging binding modes, respectively [14].

The trinuclear complex, [Fe_3_(CH_3_COO)_4_(H_2_O)_4_(L2)] (**2**), was obtained as a black solid from the reaction of H_2_L2 with [Fe(CH_3_COO)_2_] (mol ratio = 1 : 1). It was insoluble in most common organic solvents, except DMSO. As for** 1**, its structural formula was proposed based on the CHN elemental analytic data and IR spectrum, which showed peaks for coordinated H_2_O at 3296 cm^−1^, C=N at 1592 cm^−1^, ${\overset{-}{\upsilon }}_{\text{asym}}$COO at 1532 cm^−1^, ${\overset{-}{\upsilon }}_{\text{sym}}$COO at 1401 cm^−1^, and peaks for L2. The Δ value for CH_3_COO ligand was 131 cm^−1^, which suggests a chelating binding mode.

The proposed structures for both complexes were then geometry optimized, and then IR spectral simulations were performed within the domain of DFT. After geometrical optimization for** 1**, the Fe–Fe atomic distance was 3.193 Å, while the Fe–O–Fe and O–C–O (for bridging CH_3_COO) bond angles were 101.9° and 117.6°, respectively. The O–C–O bond angle for the bridging CH_3_COO is in good agreement with the theoretical value of 120° for a* sp* ^2^ hybridized carbon.

**Figure 2:**
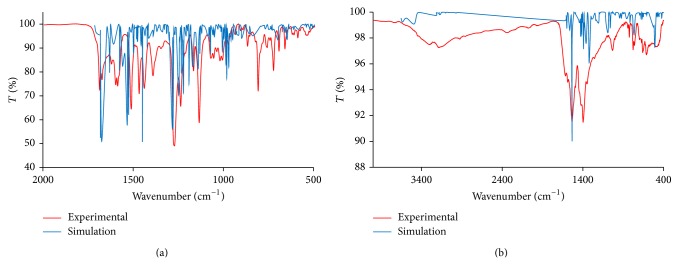
The experimental and simulated IR spectra for (a)** 1** and (b)** 2**.

**Figure 3:**
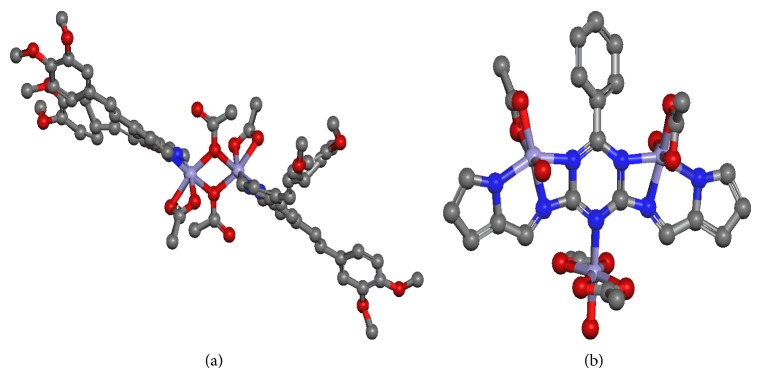
Molecular models for (a)* trans*-[Fe_2_(CH_3_COO)_4_(L3]) and (b) [Fe_3_(CH_3_COO)_4_(H_2_O)_4_(L2)] (**2**). H atoms are removed for clarity.

For IR spectral simulation, the structure for** 1** was modelled on both* cis*- and* trans*-[Fe_2_(CH_3_COO)_4_(L3)_2_], where L3 = 4,4′-bis(tetramethoxy)styryl-2,2′-bipyridine, and for both monodentate and bidentate bridging CH_3_COO. The results (Figure 2) show a better fit for* trans*-[Fe_2_(CH_3_COO)_4_(L3)_2_] with monodentate bridging CH_3_COO for** 1** and good agreement for** 2** (Figure 3). Accordingly, we are quite confident that both complexes** 1** and** 2** have the proposed structures as shown in Figure 1.

### 3.2. Electronic Absorption Spectroscopy

The electronic absorption spectrum for** 1** in CHCl_3_ shows a strong singlet metal-to-ligand charge transfer (^1^MLCT) peak at 544 nm (*ε*_max⁡_ = 2194 M^−1^ cm^−1^) and two weak* d*-*d* peaks at 1412 nm (*ε*_max⁡_ = 25.6 M^−1^ cm^−1^) and 1755 nm (*ε*_max⁡_ = 25.6 M^−1^ cm^−1^). The ^1^MLCT peak is assigned to *t*_2g_ → *π* ^*^ electronic transition for LS Fe(II) [3], while the* d-d* peaks are assigned to the ^5^T_2g_ → ^5^E_g_ electronic transition for high-spin (HS) Fe(II). The splitting of the* d-d* peak for the HS Fe(II) may be attributed to a distorted octahedral N_2_O_4_ coordination core at this centre, as a result of weaker Fe–N and Fe–O bonds [15]. Also found is a* d-d* peak for LS Fe(II), assigned for ^1^A_1g_ → ^1^T_1g_, which appeared as a shoulder on the strong MLCT peak. The spectral data suggest the presence of HS and LS Fe(II) atoms in this complex.

The electronic absorption spectrum for** 2** in DMSO shows a broad* d-d* band at 700 nm (*ε*_max⁡_ = 137 M^−1^ cm^−1^) followed by broad overlapping bands at about 340 nm (*ε* ~ 420 M^−1^ cm^−1^). These bands are assigned to ^5^T_2g_ → ^5^E_g_ for HS Fe(II), and ^1^A_1g_ → ^1^T_1g_ for LS Fe(II) [4, 8], L2^2−^, and CH_3_COO^−^ intraligand electronic transitions, respectively.

### 3.3. Magnetic Properties

**Figure 4:**
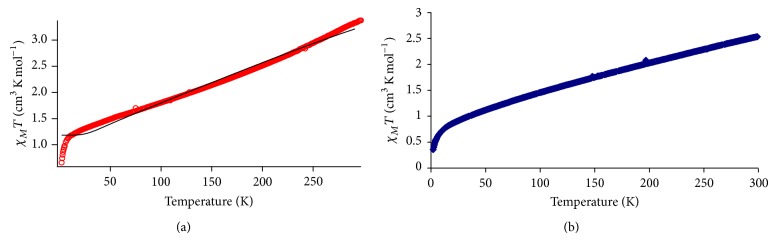
Plot of *χ*_*M*_ *T* versus *T* for (a)** 1** (circles = experimental; line = theoretical) and (b)** 2**.

The temperature-dependence magnetic susceptibilities for** 1** and** 2** were measured in the form of *χ*_*M*_ *T* versus *T* using the SQUID magnetometer. The plot for** 1** (Figure 4(a)) shows a good fit between the experimental curve, in the temperature range of about 60 K to about 250 K, with the theoretical curve obtained using the formula for a symmetrical dinuclear complex [16] and inserting the values of *g* = 1.9 and *J* = −81.2 cm^−1^ into the formula

(4) χM=Ng2β23kT∑SS(S+1)(2S+1)exp⁡⁡[−E(S)/kT]∑S(2S+1)exp⁡⁡[−S/kT],E(S)=−J2S(S+1).

The *χ*_*M*_ *T* value for** 1** decreased gradually from 3.35 cm^3^ K mol^−1^ at 294 K to 1.12 cm^3^ K mol^−1^ at 8.1 K and then rapidly to about 0.66 cm^3^ K mol^−1^ at 2 K due to the zero-field effect. From this, it can be inferred that** 1** was made up of 56.8% HS and 43.2% LS Fe(II) atoms at 294 K, since the expected *χ*_*M*_ *T* value for a dinuclear octahedral HS Fe(II) complex is 5.9 cm^3^ K mol^−1^ at this temperature [16]. It is worth noting that the *g* value for the complex (1.9) was slightly lower than the theoretical value (2.0023). This suggests an axially distorted octahedral environment, expected at the HS Fe(II) atom. The calculated value for the isotropic interaction parameter (*J* = −81.2 cm^−1^) indicates moderate antiferromagnetic interaction between the two Fe(II) centres, postulated to occur indirectly through the two monoatomic CH_3_COO bridges (superexchange pathway) [17]. Using the calculated *J* value and its correlation with the bridging angle deduced by Crawford et al., *J* (cm^−1^) = −74*α* (degrees) + 7270 [18], the calculated Fe–O–Fe bridging angle (*α*) of the dimeric complex is 99.3°, which agrees well with the value of 101.9° obtained from molecular modeling.

A plot of *χ*_*M*_ *T* versus *T* (Figure 4(b)) for** 2** shows that its *χ*_*M*_ *T* values decreased gradually from 2.5 cm^3^ K mol^−1^ at 300 K to about 0.9 cm^3^ K mol^−1^ at 25 K and then more abruptly to about 0.4 cm^3^ K mol^−1^ at 2 K. From these observations, it can be inferred that the complex was made up of 27.7% HS and 72.3% LS Fe(II) atoms at 300 K (the expected *χ*_*M*_ *T* value for a trinuclear octahedral HS Fe(II) complex at this temperature is 9.03 cm^3^ K mol^−1^) [16].

The results also indicate that the electronic configuration for HS Fe(II) in both complexes changed to LS on cooling from room temperature.

### 3.4. Photophysical Studies

Two important parameters for DSSC applications are band gap (*E*) and excited state lifetime (*τ*). Electronic spectroscopies (absorption and photoluminescence) may be used to calculate the optical band gaps (*E*_*o*_), using the equation *E*_*o*_ = *hc*/*λ*, where *h* = Planck constant (6.626 × 10^−34^ J s^−1^), *c* = velocity of light (3.0 × 10^8^ m s^−1^), and *λ* = absorption edge of charge-transfer (CT) band or emission edge. The calculated value in J was then converted to eV using the conversion factor 1 Joule = 6.24 × 10^18^ eV. The most simple and direct method used to obtain the *E*_*o*_ value is to determine the wavelength at which the extrapolation of the absorption edge crosses the baseline. The *τ* value may be calculated from the slope of the fluorescence decay curve and using the equation *F*_*t*_ = *F*_*o*_ *e* ^−*t*/*τ*^ (*F*_*t*_ = intensity at time *t*, *F*_*o*_ = intensity at initial time, *t* = time after absorption).

**Figure 5:**
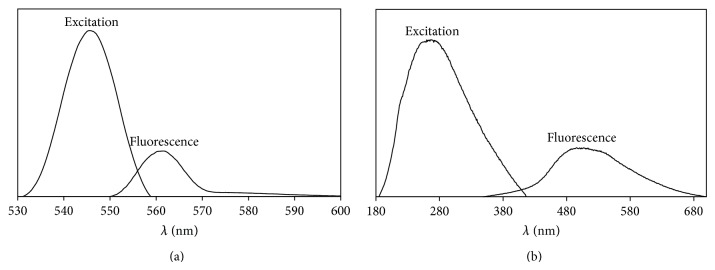
The excitation and fluorescence spectra of (a)** 1** (*λ*_ex_ = 544 nm) and (b)** 2** (*λ*_ex_ = 267 nm).

For** 1**, the *E*_*o*_ value was 1.9 eV from its absorption spectrum (*λ* = 643 nm). Upon excitation at 544 nm, its fluorescence spectrum shows a weaker peak at *λ*_max⁡_ 561 nm (Figure 5(a)). Hence, the *E*_*o*_ value was 2.2 eV (*λ* = 574 nm) and *τ* was 0.7 ns. The corresponding values for** 2** were *E*_*o*_ (absorption) = 3.5 eV (*λ* = 352 nm), *E*_*o*_ (emission after excitation at 267 nm) = 1.8 eV (*λ* = 700 nm) (Figure 5(b)), and *τ* = 2.8 ns.

Three things of interest to note for both complexes are as follows: (a) the intensities of the emission peaks were much weaker than the absorption peak. This is due to competitive radiationless process(es); (b) the *E*_*o*_ values from the absorption spectrum were lower than those from the fluorescence spectrum. The difference is due to the stabilization of HOMO of the excited Fe(III) complexes formed upon ^1^MLCT transition (a stronger Fe–N bond); and (c) the excited-state lifetime in** 1** was much shorter than** 2**, which was most likely due to the much higher excitation energy for the latter complex [2]. However, the lifetime for both complexes was significantly shorter than [Ru(2,2′-bipyridine-4,4′-dicarboxylic acid)_2_(NCS)_2_] (*τ* = 50 ns) [5], which are expected due to the low lying ligand field state in Fe(II) complexes. However, their lifetimes are sufficiently long compared to the time required for the injection of an electron into the TiO_2_ conduction band, reported to occur within femtoseconds [19].

### 3.5. Electrochemical Studies

**Figure 6:**
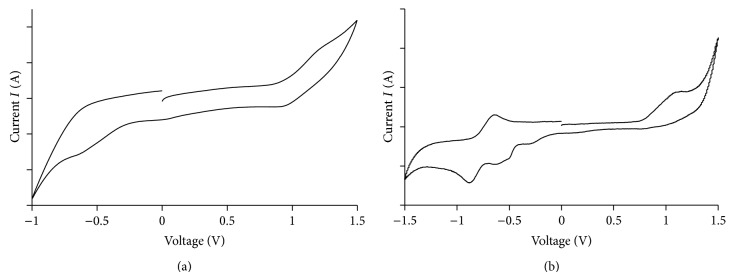
CV scans for (a)** 1** and (b)** 2**.

The CV scan for** 1** (Figure 6) shows an anodic peak at +1.2 V and two cathodic peaks at +0.05 V and −0.56 V. These peaks were assigned to the oxidation of Fe(II) to Fe(III) and reduction of Fe(III) to Fe(II) [3] and L1 to L1^−^ [20], respectively. The peaks were weak due to the presence of eight 14-carbon linear alkyl chains acting as an “insulating” layer for the transfer of electrons to/from the electrode surface [21]. The potential separation (Δ*E*) between the anodic and cathodic peaks was 1150 mV, and the cathodic-to-anodic peak current (*I*_pc_/*I*_pa_) was close to unity. These indicate a quasireversible redox process and absence of coupled chemical reaction(s), respectively [21]. The formal electrode potential for** 1** was +0.63 V (versus SCE). This was calculated using the equation *E*′(Fe^III^/Fe^II^) = (1/2)(*E*_pf_ + *E*_pr_), where *E*_pf_ = forward peak potential (+1.2 V) and *E*_pr_ = return peak potential (+0.05 V). This was comparable to the value of +0.68 V reported by Ferrere for [Fe(5,5′-dicarboxylic acid-2,2′-bipyridine)_2_(CN)_2_]) [3].

For** 2**, the CV scan (Figure 6(b)) shows an anodic peak at +1.1 V when the electrode potential was increased from 0 V to 1.5 V, followed by four cathodic peaks at −0.29 V, −0.51 V, −0.62 V, and −0.88 V when the electrode potential was reduced from +1.5 V to −1.5 V and finally an anodic peak at −0.71 V when the electrode potential was increased from −1.5 V to 0 V. Firstly, it is noted that the anodic and cathodic peaks for** 2** were more pronounced than** 1**, consistent with the absence of long alkyl chains attached to L2^2−^ ligand. Next, the value of *E*(Fe^III/II^) was +0.41 V (versus SCE), which was lower than** 1**, indicating a more facile oxidation of Fe(II). Finally, other peaks observed may be assigned to ligand-based redox processes.

The electrochemical bandgap (*E*_*e*_) may be calculated using the relationship, *E*_*e*_ = |HOMO − LUMO|, where HOMO = (onset oxidation peak voltage + 4.4) eV and LUMO = (onset reduction peak voltage + 4.4). Hence *E*_*e*_ = 0.83 eV for** 1** and *E*_*e*_ = 0.69 eV for** 2**. The higher *E*_*e*_ for** 1** is consistent with the presence of insulating alkyl groups in this complex.

## 4. Conclusions

Both dimeric (**1**) and trinuclear (**2**) complexes have LS and HS Fe(II) atoms. Complex** 1** has a lower optical bandgap, higher electrochemical bandgap, shorter excited state lifetime, and higher formal redox potential than complex** 2**. Both complexes were potential DSSC materials, but** 2** is better than** 1** based on its longer excited lifetime and lower redox potential. It can be concluded from this work that higher nuclearity Fe(II) complexes are better potential as DSSC materials, while lower MLCT transition energies led to the formation of complexes with lower optical band gaps but also shorter excited state lifetimes, and the presence of insulating long alkyl chains resulted in higher electrochemical band gaps and higher formal redox potentials.
